# Physical Organohydrogels With Extreme Strength and Temperature Tolerance

**DOI:** 10.3389/fchem.2020.00102

**Published:** 2020-03-10

**Authors:** Jing Wen Zhang, Dian Dian Dong, Xiao Yu Guan, En Mian Zhang, Yong Mei Chen, Kuan Yang, Yun Xia Zhang, Malik Muhammad Bilal Khan, Yasir Arfat, Yasir Aziz

**Affiliations:** ^1^College of Bioresources Chemical and Materials Engineering, Shaanxi University of Science & Technology, Xi'an, China; ^2^National Demonstration Center for Experimental Light Chemistry Engineering Education (Shaanxi University of Science & Technology), Xi'an, China; ^3^State Key Laboratory for Strength and Vibration of Mechanical Structures, International Center for Applied Mechanics, School of Aerospace Engineering, Xi'an Jiaotong University, Xi'an, China; ^4^Research Center for Semiconductor Materials and Devices, College of Arts and Sciences, Shaanxi University of Science & Technology, Xi'an, China

**Keywords:** organohydrogels, high strength, anti-freezing, non-drying, temperature tolerance

## Abstract

Tough gel with extreme temperature tolerance is a class of soft materials having potential applications in the specific fields that require excellent integrated properties under subzero temperature. Herein, physically crosslinked Europium (Eu)-alginate/polyvinyl alcohol (PVA) organohydrogels that do not freeze at far below 0°C, while retention of high stress and stretchability is demonstrated. These organohydrogels are synthesized through displacement of water swollen in polymer networks of hydrogel to cryoprotectants (e.g., ethylene glycol, glycerol, and d-sorbitol). The organohydrogels swollen water-cryoprotectant binary systems can be recovered to their original shapes when be bent, folded and even twisted after being cooled down to a temperature as low as −20 and −45°C, due to lower vapor pressure and ice-inhibition of cryoprotectants. The physical organohydrogels exhibit the maximum stress (5.62 ± 0.41 MPa) and strain (7.63 ± 0.02), which is about 10 and 2 times of their original hydrogel, due to the synergistic effect of multiple hydrogen bonds, coordination bonds and dense polymer networks. Based on these features, such physically crosslinked organohydrogels with extreme toughness and wide temperature tolerance is a promising soft material expanding the applications of gels in more specific and harsh conditions.

## Introduction

Hydrogels are the typical soft materials, by virtue of their great potentials in applications spanning from soft robotics, sensors, actuators to tissue engineering (Wegst et al., 2014; Iwaso et al., 2016; Kim et al., 2016; Banerjee et al., 2018; Dong et al., 2018; Hu et al., 2019). Nevertheless, conventional hydrogels are considered to be mechanically weak due to lack of an effective energy dissipation mechanism or intrinsic structural heterogeneity (Dhivya et al., 2015; Yuk et al., 2016), limiting utilization in some fields that require excellent mechanical properties (Gao et al., 2016; Fan et al., 2019; Lai et al., 2019). Therefore, improving mechanical properties of hydrogels became an important research hotspot. So far, versatile strategies to achieve tough hydrogels have been emerged, including double-network hydrogels (Gong et al., 2003; Gong, 2014; Liang et al., 2016; Chen et al., 2018; Jing et al., 2019), nanocomposite hydrogels (Haraguchi and Takehisa, 2002; Chen et al., 2015; GhavamiNejad et al., 2016; Liu Y. et al., 2017; Zhu et al., 2017), topological hydrogels (Okumura and Ito, 2001; Li et al., 2018), macromolecular microsphere composite hydrogels (Huang et al., 2007; Gu et al., 2016; Zhang and Khademhosseini, 2017; Wang Z. et al., 2018), hydrophobic association hydrogels (Li et al., 2012; Mihajlovic et al., 2017; Han et al., 2018), hydrogen bonding/dipole-dipole reinforced hydrogels (Han et al., 2012; Zhang et al., 2015; Qin et al., 2018), and many others (Gong et al., 2016; Liu J. et al., 2017; Zhao et al., 2019). However, almost all of the hydrogels swollen a large amount of water in polymer networks cannot resist a cold or hot environment (Wei et al., 2014, 2015; Wang W. et al., 2018), hindering the application of tough hydrogels in harsh conditions. Subzero temperature results in freezing of hydrogels, while high temperature lead to drying (Rong et al., 2017; Zhang et al., 2018). Freezing and drying cause the hydrogels to hard, opaque and dry, which undoubtedly change the integrated mechanical properties of hydrogels, leading to unstable nature under wide temperature range (Han et al., 2017; Lou et al., 2019). So far, it is still a challenge to design a hydrogel with enhanced and tunable mechanical strength together with extreme temperature tolerance.

Recently, two approaches have been proposed to develop hydrogels with extreme temperature tolerance. One is introduction of an ionic compound (e.g., NaCl, LiCl, and CaCl_2_) to hydrogels, i.e., the polymer networks swollen with salt water (Morelle et al., 2018), for dropping the ice point of water according to the principle of colligative properties of solution. However, water can be evaporated from polymer networks under high temperature causing unstable mechanical properties. The other strategy is the introduction of a water-cryoprotectant binary solvent system into organohydrogel (OHG) networks through synthesis or displacement (Gao et al., 2017; Rong et al., 2018). Compared with the hydrogel containing ionic compound, water-cryoprotectant binary solvent endows stable mechanical properties to gels under both low and high temperature, due to the advantages of cryoprotectants including relatively high volatile point and inhibition ice crystallization (Elliott et al., 2017). The cryoprotectants, including ethylene glycol (EG), glycerol (GC), and d-sorbitol (SB) are suitable choices for fabricating organhydrogels swollen water-cryoprotectant binary solvents, which was firstly reported by Wang's group (Shi et al., 2017).

In the present study, we found that the mechanical properties and temperature tolerance could be dramatically enhanced by fabricating tough physically crosslinked organhydrogels *via* solvent displacement approach. The tough organohydrogels were prepared through displacing cryoprotectants (i.e., EG, GC, SB) into our previously reported Eu-alginate/PVA hydrogel networks mainly crosslinked by hydrogen bonds formed among PVA polymers and coordination bonds between Na-alginate networks and Eu^3+^ ions (Wang et al., 2015; Hu et al., 2017). Multiple hydrogen bonds forming among cryoprotectants and PVA polymers enhance mechanical properties of organohydrogels. Moreover, cryoprotectants disrupt the formation of ice crystal lattices of the residual water, endowing extreme toughness and temperature tolerance to the organohydrogels. Furthermore, tunable mechanical performance of the oganohydrogels can be controlled by either selecting cryoprotectants or by varying the extent of solvent displacement. Therefore, physically crosslinked organohydrogels with enhanced and tunable mechanical properties, as well as extreme temperature tolerance could be designed and synthesized, potentially expanding scientific research and practical applications of gels.

## Experiment

### Materials and Methods

Polyvinyl alcohol (PVA, Mn = 205,000) and sodium alginate (Na-alginate) were purchased from Sigma-Aldrich (Shanghai, China). Alginate is a linear copolymer of α-L-guluronic acid (G unit) and β-D-mannuronic acid (M unit). Europium chloride hexahydrate (EuCl_3_·6H_2_O) was obtained from Qufu Chemical Co. Ltd. (Qufu China). Ethylene glycol, glycerol, and d-sorbitol were supplied by Cheng Jie Chemical Engineering Co. Ltd. (Shanghai, China). All chemicals were received and used without further purification. Ultrapure water with a resistivity higher than 18.2 MΩ·cm was supplied by a Millipore Simplicity 185 system, which was deoxygenated three times by using a freeze-pump-thaw method before use.

### Preparation of Eu-Alginate/PVA Hydrogel and Organohydrogels

Eu-alginate/PVA hydrogel was prepared by following the method described in our previous work (Hu et al., 2017). Briefly, Na-alginate and PVA were dissolved in ultrapure water to produce a homogeneous solution, wherein the molar ratio of Na-alginate and PVA is 1:9. The Na-alginate/PVA hydrogel was then obtained by two freeze/thaw cycles of the polymer solution. Subsequently, the Na-alginate/PVA hydrogel was soaked into the aqueous solution of EuCl_3_·6H_2_O (0.01 mol/L), obtaining Eu-alginate/PVA hydrogel.

**Figure 1:**
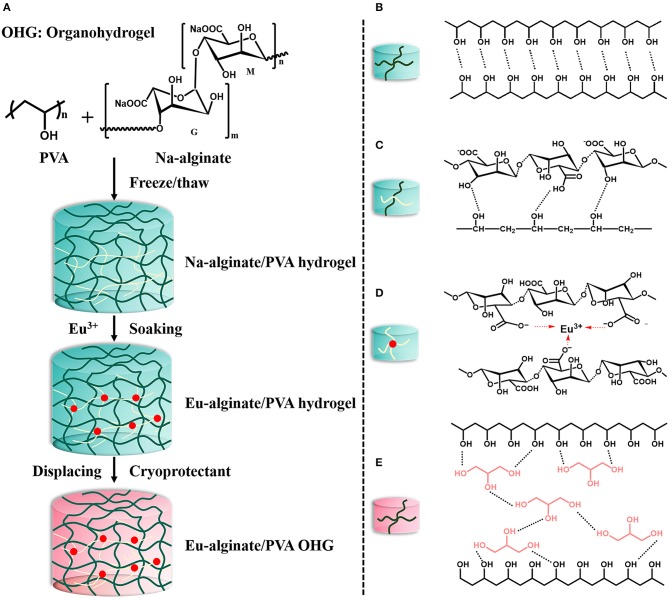
The design strategy for physical organohydrogels with enhanced strength and extreme temperature tolerance *via* solvent displacement method. **(A)** Synthesis procedures and proposed structure of Eu-alginate/PVA organohydrogels. **(B)** Schematic illustration of hydrogen-bonding interactions of adjacent PVA polymers chains. **(C)** Hydrogen bonds between PVA polymers and Na-alginate macromolecules. **(D)** Anionic carboxyl groups in alginate structure coordination with Eu^3+^ ions **(E)**. Hydrogen bonding between glycerol (GC) and PVA polymers in OHG_*GC*_ obtained by solvent displacement method.

Eu-alginate/PVA organohydrogels were synthesized by using solvent displacement method (Chen et al., 2018). Herein, Eu-alginate/PVA hydrogel was directly placed into a vessel containing three different cryoprotectant solutions, namely ethylene glycol (EG), glycerol (GC) solution, and d-sorbitol (SB) aqueous solution (SB: H_2_O = 2:1), respectively. For the sake of brevity, we denote these Eu-alginate/PVA organohydrogels as OHG_*EGt*_, OHG_*GCt*_, and OHG_*SBt*_. OHG refers to organohydrogels, EG, GC, and SB denote the corresponding solution, and t represents displacement time. To estimate the solvent displacement behaviors, the weight ratio (W_a_/W_b_, where W_b_ and W_a_ refers to the weight before and after solvent displacement, respectively) of the organohydrogels was calculated. OHG_0_ means original Eu-alginate/PVA hydrogel. The synthesis procedures and structure of Eu-alginate/PVA OHG were illustrated in Figure 1.

### Measurement of Mechanical Properties

All mechanical properties of the gels were tested on a tensile tester (CMT6503, MTS, United States). Tensile test was performed under room temperature, by setting a 500 N sensor. All the samples were cut into dumbbell-shaped with the help of a caliper in the size of tensile part 2 × 2 × 12 mm (Hengliang Liangju Co. Ltd., Shanghai, China). Both ends of the dumbbell-shaped samples were connected to the clamps. The upper clamp was pulled by the load cell at a constant velocity of 100 mm min^−1^ while the lower clamp was fixed. From the stress-strain curve, the stress, Young's modulus and fracture strain of those gels can be calculated. The Young's modulus (E) could be calculated by τ = stress/strain when strain is lower than 10%, where stress represents the force causing the deformation divided by the area to which the force is applied, and strain denotes the ratio of the change in elongation compared to the original length of the sample. Since strain is a dimensionless quantity, the unit of E is same as that of stress. Fracture strain is the maximum deformation tensile length that an object or substance can withstand, which can be calculated by ε = *(L–L*_0_*)/L*_0_, where L_0_ and L is the original and deformation length of the sample, respectively.

### Characterization of Non-drying and Anti-freezing Properties

To gain further insight into the non-drying property of organohydrogels, the weight rate (W_t_/W_0_) was calculated. The initial weight of the sample was recorded as W_0_. W_t_ denotes the weight of the corresponding sample heated with different displacement times. The organohydrogels were heated at the temperature of 50°C. Characterization of the anti-freezing property of organohydrogel was carried out by freezing the organohydrogels at the temperature of −20°C or even −45°C for 2 h. The frozen organohydrogels were quickly folded or twisted and then left to recover freely. After 5 min, the anti-freezing property of the organohydrogels was illustrated in the digital pictures.

### Structural Characterization

Characterization of the gels including morphology, composition, and crystalline structure, was carried out to further understand the solvent displacement mechanism. The morphology of the organohydrogel samples was visualized using a field-emission scanning electron microscopy (SEM, JROL JSM-7000F, Japan). Fourier transform infrared (FTIR) spectrum was collected at ambient temperature using a Nicolet 5700 FTIR spectrometer (Thermo Scientific, United States) over a wavelength ranges from 400 to 4,000 cm^−1^ after 64 scans at 2 cm^−1^ resolution. X-ray diffraction (XRD) patterns of the gels were obtained at room temperature on a Philips X'Pert pro MPD diffractometer, using Cu-Kα radiation (λ = 1.5406 Å) in the range of 2θ = 10–90° and the scanning rate was set at 0.02°/s. The hydrogel and organohydrogels were freeze-dried, before characterizing by the SEM and FTIR. The XRD results were directly obtained from the as-prepared hydrogel and organohydrogels.

## Results and Discussions

### Synthesis of the Eu-Alginate/PVA Organohydrogels

The main synthetic procedures including three sequential steps to obtain the Eu-alginate/PVA organohydrogels were shown in Figure 1A. Firstly, homogeneous solution of PVA and Na-alginate was freezed/thawed for two cycles to obtain Na-alginate/PVA hydrogel. The procedure facilitates the formation of hydrogen bonds between the polymer chains in the Na-alginate/PVA hydrogel. The hydrogen bonds formed between hydroxyl groups (–OH) of PVA polymers (Figure 1B) as well as between carboxyl groups (–COOH) of Na-alginate macromolecules and the hydroxyl groups of PVA polymers in the crosslinked nodes (Figure 1C). Subsequently, Na-alginate/PVA hydrogel was immersed in EuCl_3_ solution, and Eu^3+^ ions are easily accessible to anionic carboxyl groups in alginate structure center to form coordinate bonds (Figure 1D). Eu^3+^ ions, with low toxicity and antibacterial property, not only provide the photoluminescent property but also serve as physical crosslinkers for Na-alginate. Furthermore, coordination bonds between trivalent Eu^3+^ ions and the carboxyl ligands of Na-alginate act as physical sacrificial bonds for energy dissipation, leading to good mechanical property. A tough Eu-alginate/PVA hydrogel could be obtained, and the hydrogel exhibits a dual physically crosslinked polymer networks including hydrogen bonds forming between polymer chains, as well as coordination bonds between Eu^3+^ ions and –COO^−^ groups, while the dual crosslinked polymer networks endow tough mechanical behavior to the hydrogel (Hu et al., 2017).

Then, the Eu-alginate/PVA hydrogel was soaked into cryoprotectant solutions for a certain time to obtain organohydrogels swollen water-cryoprotectant binary solvent of EG, GC, and SB, respectively. Owing to osmotic pressure, a large amount of water in the hydrogel networks was displaced by cryoprotectants (bottom in Figure 1A). Furthermore, based on the principle of dissolution in a similar material structure, cryoprotectant molecules containing hydroxyl groups could be dispersed well in polymer networks to form hydrogen bonds with PVA polymer chains, obtaining the final extreme tough and temperature tolerant organohydrogels denoted as OHG_*EG*_, OHG_*GC*_, and OHG_*SB*_. We anticipate that the organohydrogels exhibit the superior mechanical properties in virtue of multiple hydrogen bonds between cryoprotectant molecules and PVA polymer chains (Figure 1E, taking OHG_*GC*_ as an example).

**Figure 2:**
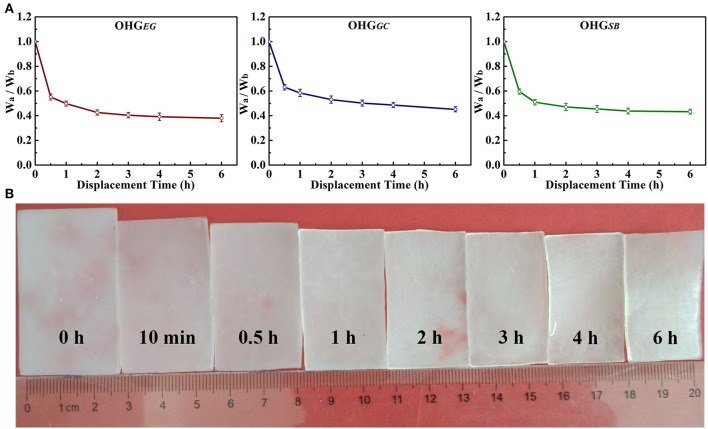
Weight rate (W_a_/W_b_) of the organohydrogels **(A)**. W_a_ denotes the weight of the gel immersed in different cryoprotectant solution [ethylene glycol (EG), glycerol (GC), and d-sorbitol (SB) solutions] with different displacement time. W_b_ represents the original weight of the Eu-alginate/PVA hydrogel. **(B)** Digital pictures show change in the size of OHG_*EG*_ with prolonged displacement time of 0 h, 10 min, 0.5 h, 1 h, 2 h, 3 h, 4 h and 6 h.

Figure 2 shows the weight rate (W_a_/W_b_, where W_b_, W_a_ represents the weight before and after solvent displacement, respectively) of organohydrogels with the displacement time ranging from 0.5 to 6 h. The weight rate is dependent on displacement time of the cryoprotectants. As can be seen, with increased displacement time, the weight rate of the organohydrogels decreased and finally almost reached a displacement equilibrium state. Especially, the weight rate (W_a_/W_b_) of OHG_*EG*_, OHG_*GC*_, and OHG_*SB*_ all decreased quickly at initial 0.5 h, from 1 to 0.55, 0.63, and 0.58, respectively (Figure 2A). The water in the hydrogel system usually exists in three states, i.e., free water, intermediate water, and nonrotational bound water (Cerveny et al., 2005; Wu et al., 2019). The fast-decreased weight rate is attributed to the fact that most of the “free water” in hydrogel networks is displaced quickly due to unbound water molecules. However, the intermediate water weakly interacted with the polymer networks is displaced slowly with the cryoprotectant molecules. Moreover, it is difficult for the strongly bound water to undergo solvent displacement. As an example, with the displacement time ranging from 0 to 6 h, the fast shrank and decreased volume of the ethylene glycol based organohydrogel (OHG_*EG*_) could be visualized at initial time (<0.5 h) and then it slowed down (Figure 2B). These results indicate a successful solvent displacement between the water and cryoprotectant molecules. With this approach, the Eu-alginate/PVA hydrogel was transformed into Eu-alginate/PVA organohydrogels with dense polymer networks swollen water-cryoprotectant binary solvent, leading to enhanced capabilities of mechanical properties, moisture holding and temperature tolerance.

### Mechanical Properties of the Eu-Alginate/PVA Organohydrogels

**Figure 3:**
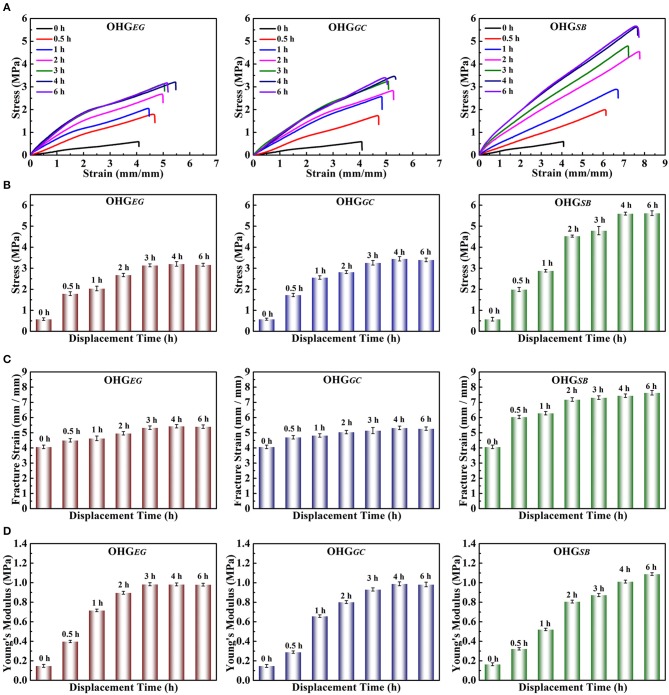
Mechanical properties of the Eu-alginate/PVA organohydrogels (OHG_EG_, OHG_GC_, OHG_SB_) treated with cryoprotectants for different times. **(A)** stress-strain curves, **(B)** stress-displacement time histogram, **(C)** Fracture strain-displacement time histogram, **(D)** Young's modulus-displacement time histogram.

The effects of cryoprotectants on the mechanical properties of organohydrogels were tested by tensile experiments. Figure 3 shows the mechanical properties (tensile strength, fracture strain and Young's modulus) of the organohydrogels (OHG_*EG*_, OHG_*GC*_, and OHG_*SB*_) displaced by three different cryoprotectants. The tensile strength (Figure 3B), fracture strain (Figure 3C), and Young's modulus (Figure 3D) of the organohydrogels were higher than that of original Eu-alginate/PVA hydrogel, which can be ascribed to the synergistic effect of the multiple hydrogen bonds and the dense polymer networks. For instance, the tensile strength of OHG_*SB*_ increases from 0.58 ± 0.06 MPa to as high as 5.62 ± 0.41 MPa, fracture strain raised from 4.07 ± 0.04 to as high as 7.63 ± 0.02 and Young's modulus ascended from 0.16 ± 0.01 to 1.08 ± 0.03 MPa as the displacement time gradually increased to 6 h. The tensile strength is higher than many of the previously reported organohydrogels, such as polydopamine decorating carbon nanotubes (PDA-CNT)/copolymer of acrylamide (AM) and acrylic acid (AA) (PAM-co-PAA) organohydrogel (0.07 MPa stress, 7.01 strain, Han et al., 2017), PVA/poly(3,4-ethylenedioxythiophene):polystryrene sulfonate (PEDOT:PSS) organohydrogel (2.1 MPa stress, 7.60 strain, Rong et al., 2017), and gelation organohydrogel (2.06 MPa stress, 6.88 strain, Qin et al., 2019), as shown in Figure S1. The dramatic enhancement in mechanical properties of the organohydrogels is directly related to crosslinking density, which dominated by the largely increased hydrogen bonds between the cryoprotectant molecules and polymer chains in the organohydrogels (Pan et al., 2018). Interestingly, the tensile strength and the Young's modulus of OHG_EG_ and OHG_GC_ increased by increasing displacement time and then its tended to balance. The tensile strength of OHG_*EG*_ and OHG_*GC*_ prepared at displacement time of 3 and 4 h reached to 3.20 ± 0.37 and 3.45 ± 0.42 MPa, respectively. The Young's modulus of the organohydrogels reached to 0.98 ± 0.34 and 0.99 ± 0.42 MPa, respectively. The excellent tensile strength and Young's modulus achieved in the shorter displacement time could be attributed to the smaller molecules of EG and GC than SB. Overall, based on solvent displacement method, the mechanical strength of the physically crosslinked organohydrogels can be dramatically enhanced. In addition, the types of cryoprotectants and displacement time play important roles in controlling mechanical performances of organohydrogels to fulfill the requirements in specific potential applications.

### The Non-drying and Anti-freezing Properties of the Eu-Alginate/PVA Organohydrogels

**Figure 4:**
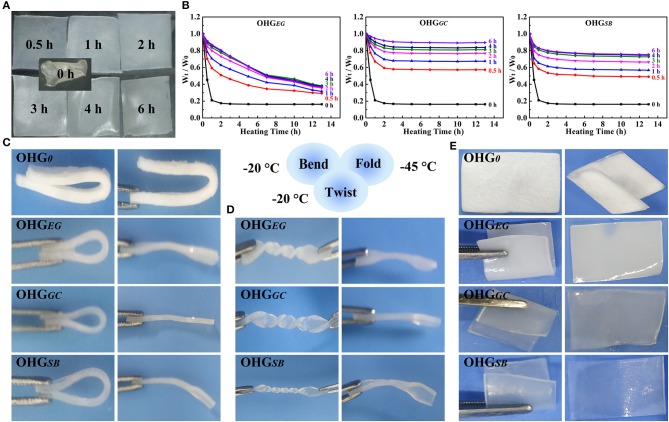
Non-drying **(A,B)** and anti-freezing **(C–E)** properties of the organohydrogels. **(A)** Digital pictures of the organohydrogels soaked in GC solution for different times (0, 0.5, 1, 2, 3, 4, 6 h) and then heated in a vacuum oven at 50°C until constant weight was gained. **(B)** Weight retention rate of OHG_*EG*_, OHG_*GC*_, and OHG_*SB*_ with different immersion time under heating at 50°C for different heating time. *W*_0_ is the initial weight of the organohydrogels (OHG_0_), while *W*_t_ denotes the weight of the samples heated for different times at the temperature of 50°C. The mechanical deformation behaviors (**C**, bent; **D**, twisted for 3 × 360°; **E**, fold) and corresponding recovery state (after free recovery for 5 min) of the organohydrogels soaked for 4 h and then cooled at −20°C **(C,D)** and −45°C **(E)**.

To demonstrate the organohydrogels with a temperature tolerance (−45–50°C), we investigated the non-drying and anti-freezing properties of the organohydrogels (OHG_*EG*_, OHG_*GC*_, OHG_*SB*_), as shown in Figure 4. Firstly, to demonstrate non-drying property, the organohydrogels immersed in EG, GC, and SB for different time (0–6 h) were heated at the temperature of 50°C (0–13 h). The weight rate was calculated by (W_t_/W_0_), where W_0_ and W_t_ denotes for the weight of organohydrogels before heating and heating for certain time, respectively (Figure 4B). The weight of organohydrogels decreased by increasing heating time and then it tended to balance, due to that remaining water was evaporated from the organohydrogels. Furthermore, the organohydrogels treated with long displacement time showed high weight rate (W_t_/W_0_). Notably, it was found that the weight rate of the OHG_*GC*_ at the displacement time of 6 h exhibited the highest weight rate (over 0.9), because lower vapor pressure (compared to glycol) and fast exchange kinetics of glycerol (small molecular size compared to sorbitol) (Rajan and Matsumura, 2018). In contrast, the original hydrogel showed the lowest weight rate (0.18), indicating that OHG_0_ does not show non-drying ability due to volatilization of water.

In addition, the anti-freezing properties of the organohydrogels, i.e., deformation behaviors (c, bend; d, twist for 3 × 360°; e, fold) and corresponding recovery states were demonstrated in Figure 4. The behaviors of the organohydrogels and hydrogel under the sub-zero temperature were obviously different. The organohydrogels exhibited outstanding deformation behavior, but the original hydrogel could not recover after bending under the sub-zero temperature. The frozen hydrogel (OHG_0_) displayed a non-transparent and white morphology due to formation of an aggregate of ice crystals in the polymer networks (Figure 4C). The organohydrogels displaced by different cryoprotectants for 4 h, for example, OHG_*EG*4_, OHG_*GC*4_, and OHG_*SB*4_, showed good recovery behaviors after being bent and twisted for 3 × 360° at −20°C (Figure 4D). To further demonstrate the anti-freezing property of the organohydrogels, the organohydrogels and hydrogel were placed in a harsh condition (−45°C). In fact, the hydrogel became rigid and fragile owing to being frozen and even generated cracks on the surface during folding under the sub-zero temperature. In contrast, the organohydrogels (OHG_EG4_, OHG_GC4_, and OHG_SB4_) could return to their initial states after being bent and folded (Figure 4E). The excellent anti-freezing ability is due to the ice-inhibiting effect of cryoprotectants disrupting the formation of ice crystal lattices of the residual water molecules. The results demonstrate that the cryoprotectant based organohydrogels exhibit excellent non-drying and anti-freezing property, indicating the potential applications under a broad temperature range.

### The Microstructure of Organohydrogels and Hydrogel

**Figure 5:**
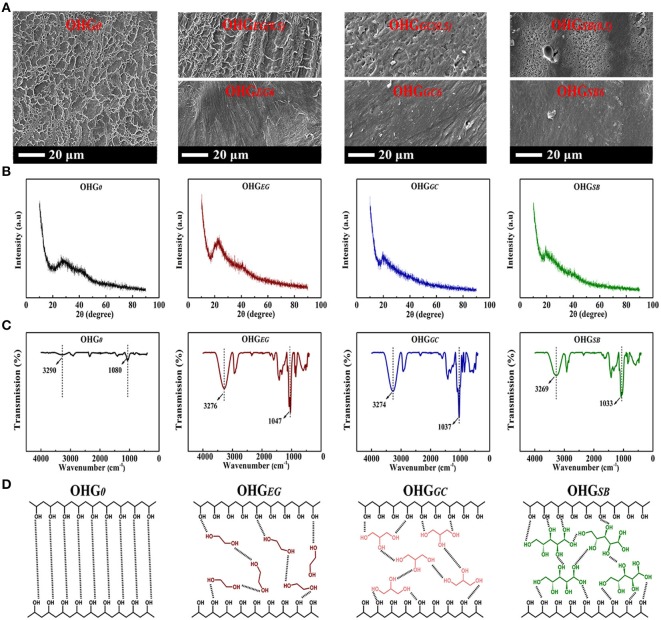
**(A)** SEM images of the gels [OHG_0_, OHG_EG(0.5)_, OHG_GC(0.5)_, OHG_SB(0.5)_, OHG_EG6_, OHG_GC6_, OHG_SB6_], **(B)** XRD patterns and **(C)** FTIR spectra of the gels (OHG_0_, OHG_*EG*6_, OHG_*GC*6_, OHG_*SB*6_). For example, OHG_EG(0.5)_ represents Eu-alginate/PVA hydrogels soaking in ethylene glycol (EG) for 0.5 h, while OHG_SB6_ denotes the hydrogels soaking in d-sorbitol (SB) for 6 h. **(D)** Schematic illustrations of the hydrogen bonds (black dotted line) between PVA polymer chains in hydrogel (OHG_0_), and EG, GC, SB molecules bridged PVA chains via forming hydrogen bonds in the corresponding organohydrogels.

To further demonstrate the effect of microstructural changes of organohydrogels and hydrogel on their performances, the SEM, XRD, and FTIR analyses were performed, respectively. As shown in the SEM images (Figure 5A), the original hydrogel (OHG_0_) displayed a distinct porous structure with loose texture, because water molecules form a lot of ice crystals under subzero temperature, and leading to porous structure mainly occupied by water after sublimation of ice crystals from the hydrogel processed by vacuum freeze-drying (Ricciardi et al., 2004). On the other hand, organohydrogels after 0.5 h displacement, i.e., OHG_EG(0.5)_, OHG_GC(0.5)_, and OHG_SB(0.5)_ presents dense structure after same treatment processes, because cryoprotectants prevent formation of ice crystals. The organohydrogels with dense structure correspond to volume shrinkage of organohydrogels because hydrophilic polymers do not swell well in the cryoprotectants (Figure 2).

Additionally, the unique microstructures could strengthen the crystallization among PVA polymer chains. It could be verified by XRD patterns where the crystal peak of PVA became more intense in the organohydrogels (OHG_*EG*(0.5)_, OHG_*GC*(0.5)_, and OHG_*SB*(0.5)_), than that of hydrogels (Figure 5B). The Eu-alginate/PVA hydrogel and organohydrogels do not show sharp crystalline diffraction peaks of PVA, because the presence of Na-alginate and Eu^3+^ ions inhibit the crystallization of PVA (Hu et al., 2017). The Eu-alginate/PVA hydrogel has a halo centered at 2θ ≈ 28° (Figure 5B) in the diffraction of pure water same as previous report (Ricciardi et al., 2004). The Eu-alginate/PVA hydrogel possesses a water content high enough to 85% while a low PVA content of about 13.5%, indicating crystalline diffraction peaks of PVA (2θ ≈ 19.4 and 20°) might be covered by the aforementioned diffraction of free pure water. After the hydrogel was transformed into organohydrogels, typical reflections of crystalline atactic PVA, with a maximum 2θ angles of 22.3, 20.1, 19.6° presents for OHG_EG_, OHG_GC_, and OHG_SB_ sample, respectively (Ricciardi et al., 2004). A slight shift of the peak around 2θ = 20.0° of three type organohydrogels could be assigned to the hydrogen bonds between PVA polymers and the cryoprotectants with different molecular structures (Zhao et al., 2019). The results indicate more crystalline PVA aggregates are formed in the organohydrogels due to the decreased relative amount of “free water,” whereas a lot of swollen amorphous PVA polymer chains present in the hydrogel. The multiple hydrogen bonds including PVA crystalline domains act as knots of the gel network, promoting the enhancement of mechanical properties (Figure 5D).

As shown in the FTIR spectrums (Figure 5C), the FTIR spectrum of Eu-alginate/PVA hydrogel shows the characteristic stretching bands of –OH at 3,290 cm^−1^ and C–O at 1,080 cm^−1^. While for the Eu-alginate/PVA organohydrogels (OHG_EG_, OHG_GC_ and OHG_SB_), the characteristic stretching band of –OH shifted to 3,276, 3,274, and 3,269 cm^−1^, respectively, as well as the characteristic stretching band of C–O shifted to 1,047, 1,037, and 1,033 cm^−1^, respectively. The shift of IR absorption bands to lower wave numbers suggests the formation of stronger H-bonding in the organohydrogels.

Based on the above analysis, schematics illustrating the interaction among PVA polymer chains and cryoprotectant molecules were presented in Figure 5D. After cryoprotectant displacement, the EG, GC, SB molecules could bridge PVA chains *via* abundant hydrogen bonds forming between cryoprotectants and PVA polymer chains. And the pivotal roles of cryoprotectant molecules can be attributed to three parts, that are, (i) Enhancing the mechanical properties of organohydrogels. (ii) Restricting volatilization of the residual water to promote non-drying ability. (iii) Disrupting the formation of ice crystal lattices as well as reducing the freezing point of H_2_O, both phenomena increase the anti-freezing capacity of organohydrogels. As a result, physically crosslinked organohydrogels with enhanced mechanical properties and extreme temperature tolerance could be designed and obtained by cryoprotectants displacement method.

## Conclusions

In summary, physically crosslinked organohydrogels with toughness and extreme temperature tolerance were successfully fabricated by solvent displacement method. Each component of the Eu-alginate/PVA organohydrogels serves the respective role for endowing excellent integrated properties. The PVA is responsible for gel backbone, offering a certain mechanical strength facilitating hydrogen bonds formation, while alginate enables the enhanced mechanical performance of the gels by coordination with Eu^3+^ ions, and interlaces with PVA polymer chains via hydrogen bonds. More importantly, cryoprotectants disrupt the formation of ice crystal lattices of water molecules. This disruption is responsible for bridging of PVA chains through abundant and stable multiple hydrogen bonds, profiting effective energy dissipation, and restricting volatilization of the residual water. The organohydrogels feature enhanced and tunable mechanical capacity, as well as freezing/heating tolerance, potentially to be used in various fields, such as medical devices, flexible electronics, and stretchable devices.

## Data Availability Statement

All datasets generated for this study are included in the article/Supplementary Material.

## Author Contributions

YC put forward the ideas about this research and designed the experiments. JZ, DD, and EZ prepared the main materials, and completed the structural characterization and performance testing of materials. JZ and DD analyzed the data and wrote the manuscript. XG, YZ, KY, MK, YAr, and YAz revised and edited the manuscript.

### Conflict of Interest

The authors declare that the research was conducted in the absence of any commercial or financial relationships that could be construed as a potential conflict of interest.
