# Corrigendum: Physical Organohydrogels With Extreme Strength and Temperature Tolerance

**DOI:** 10.3389/fchem.2020.00445

**Published:** 2020-05-25

**Authors:** Jing Wen Zhang, Dian Dian Dong, Xiao Yu Guan, En Mian Zhang, Yong Mei Chen, Kuan Yang, Yun Xia Zhang, Malik Muhammad Bilal Khan, Yasir Arfat, Yasir Aziz

**Affiliations:** ^1^College of Bioresources Chemical and Materials Engineering, Shaanxi University of Science & Technology, Xi'an, China; ^2^National Demonstration Center for Experimental Light Chemistry Engineering Education (Shaanxi University of Science & Technology), Xi'an, China; ^3^State Key Laboratory for Strength and Vibration of Mechanical Structures, International Center for Applied Mechanics, School of Aerospace Engineering, Xi'an Jiaotong University, Xi'an, China; ^4^Research Center for Semiconductor Materials and Devices, College of Arts and Sciences, Shaanxi University of Science & Technology, Xi'an, China

**Keywords:** organohydrogels, high strength, anti-freezing, non-drying, temperature tolerance

In the published article there was an error in the Funding statement. The correct number for the Key Research and Development Program of Shaanxi is “2020KWZ-006.” The correct number for the Xi'an Weiyang District Science and Technology Fund is “201927.”

The authors apologize for this error and state that this does not change the scientific conclusions of the article in any way. The original article has been updated.

