# Supplementary material for: Physical Organohydrogels With Extreme Strength and Temperature Tolerance
Source: Front Chem. 2020 Mar 10;8:102. doi: 10.3389/fchem.2020.00102 (PMC7076117; doi:10.3389/fchem.2020.00102)
Supplement: Supplementary file 1 [file Table_1.DOCX]

Supplementary Material

# The mechanical properties reported organohydrogels


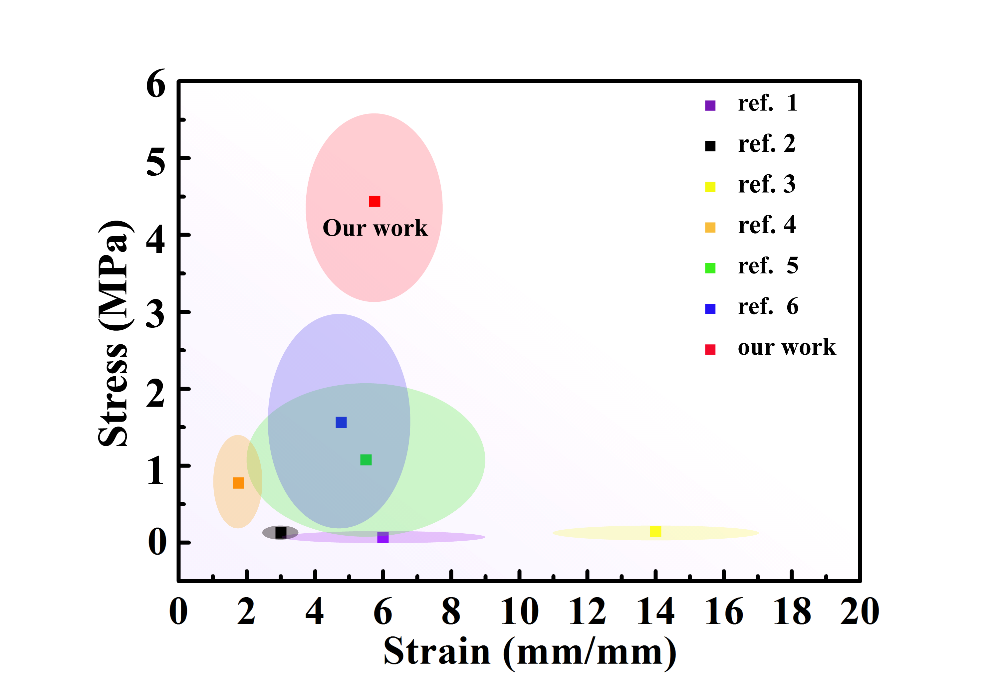


**Supplementary Figure 1.** Summary of the stress-strain curves of reported organohydrogels.

**References**

[1]Han, L., Liu, K., Wang, M., Wang, K., Fang, L., Chen, H., et al. (2017). Mussel-Inspired Adhesive and Conductive Hydrogel with Long-Lasting Moisture and Extreme Temperature Tolerance. Adv. Funct. Mater. 28(3): 1704195. doi:10.1002/adfm.201704195

[2]Rong, Q., Lei, W., Huang, J., and Liu, M. (2018). Low Temperature Tolerant Organohydrogel Electrolytes for Flexible Solid-State Supercapacitors. Adv. Energy Mater. 1801967. doi:10.1002/aenm.201801967

[3]Chen, F., Zhou, D., Wang, J., Li, T., Zhou, X., Gan, T., et al. (2018). Rational Fabrication of Anti-Freezing Non-Drying Tough Organohydrogels by One-Pot Solvent Displacement. Angew. Chem. Int. Ed. 130, 1-5. doi:10.1002/anie.201803366

[4]Lou, D., Wang, C., He, Z., Sun, X., Luo, J., and Li, J. (2019). Robust organohydrogel with flexibility and conductivity across the freezing and boiling temperatures of water. Chem. Commun. 55(58), 8422-8425. doi:10.1039/c9cc04239c

[5]Rong, Q., Lei, W., Chen, L., Yin, Y., Zhou, J., and Liu, M. (2017). Anti-freezing Conductive Self-healing Organohydrogels with Stable Strain-Sensitivity at Subzero Temperatures. Angew. Chem. Int. Ed. 56, 14159-14163. doi:10.1002/anie.201708614

[6]Qin, Z. H., Dong, D. Y., Yao, M. M., Yu, Q. Y, Sun, X., Guo, Q., et al. (2019). Freezing-Tolerant Supramolecular Organohydrogel with High Toughness, Thermoplasticity, and Healable and Adhesive Properties. ACS. Appl. Mater. Interfaces 11, 21184-21193. doi:10.1021/acsami.9b05652
